# Supplementary material for: Readability, quality, and reliability of AI-generated ınformation on myofascial pain syndrome: A comparative analysis of ChatGPT, Gemini, and Perplexity
Source: PLoS One. 2026 Jun 4;21(6):e0350402. doi: 10.1371/journal.pone.0350402 (PMC13235886; doi:10.1371/journal.pone.0350402)
Supplement: Attachment — Submitted filename: Response to reviewers.docx [file pone.0350402.s002.docx]

**Dear Editor, Thank you very much for giving us the opportunity to revise our manuscript. I would like to thank the editor and the reviewer for their valuable comments. We have now carefully revised our manuscript according to the comments as indicated below.**

Reviewer #1: This is a well-structured and timely manuscript. The topic is relevant, the methodology is reasonably rigorous, and the findings have clear clinical implications. Below is a constructive, peer-review-style critique, organized by section, with specific strengths, weaknesses, and suggestions for improvement.
Overall Assessment
1. Abstract & Material and Methods :Critical Error in Model Version Reporting
• Issue: You state you used "ChatGPT (GPT-5.2)" as of December 9, 2025. GPT-5.2 does not exist as of March 2026. OpenAI has released GPT-4, GPT-4 Turbo, GPT-4o, and o1-series models, but not GPT-5.2. This is a serious factual error that undermines credibility.
• Recommendation: Immediately verify and correct the model version. Most likely you used GPT-4 or GPT-4 Turbo. If you used a version released in late 2025, specify the exact model name (e.g., "GPT-4o-2024-11-20").

**Dear Reviewer, thank you for your kind contribution. Artificial intelligence technology is advancing rapidly, with new models being released every day. We have therefore designed our work to mature as quickly as possible and include the most up-to-date models. Below, we have organized the ChatGPT models, including their dates and models from 2018 to 2026. As can be understood from this, the 5.2 model from December 2025 was used in our study.**

***Chronological Order of ChatGPT Models (2018–2026)***

- ***GPT-1 (June 2018):*** *Introduced the Transformer architecture.*
- ***GPT-2 (February 2019):*** *Increased parameters to 1.5 billion, demonstrated improved text generation.*
- ***GPT-3 (June 2020):*** *A massive leap to 175 billion parameters, enabling foundational Few-Shot learning.*
- ***ChatGPT/GPT-3.5 (November 2022):*** *The public launch of ChatGPT, fine-tuned specifically for conversation.*
- ***GPT-4 (March 2023):*** *Multimodal capabilities introduced, allowing image input.*
- ***GPT-4 Turbo (Late 2023/Early 2024):*** *Faster, cheaper, and increased knowledge cutoff.*
- ***GPT-4o ("Omni") (May 2024):*** *Native multimodal capabilities (text, audio, vision) and significantly improved speed.*
- ***GPT-4o mini (July 2024):*** *Small, efficient model replacing GPT-3.5.*
- ***o1 ("Strawberry") (September 2024):*** *Reasoning-focused models designed for complex math/science.*
- ***GPT-4.5 (February 2025):*** *Increased parameters and enhanced "EQ" for creative writing.*
- ***GPT-4.1 / o3 / o4-mini (April 2025):*** *Continued refinement of reasoning and specialized coding capabilities.*
- ***GPT-5 / GPT-5 Thinking (August 2025):*** *The next major flagship, combining fast "Instant" responses with deeper "Thinking" reasoning.*
- ***GPT-5.1 (November 2025):*** *Personality system prompt updates ("friendly", "efficient", "cynical").*
- ***GPT-5.2 / GPT-5.2 Thinking (December 2025):*** *Incremental update focusing on reasoning speed and competitive multimodality.*
- ***GPT-5.3-Codex (February 2026):*** *Combined general intelligence and specialized coding agent.*
- ***GPT-5.3 Instant (March 2026):*** *Current default workhorse for everyday tasks.*
- ***GPT-5.4 / Thinking / Pro / Mini (March 2026):*** *Current frontier models with superior agentic (computer-use) capabilities and 1M token context.*
- ***GPT-5.5 (April 2026):*** *Latest refinement focused on agentic workflows and deep reasoning. [*[*1*](https://help.openai.com/en/articles/9624314-model-release-notes)*,* [*2*](https://ttms.com/chat-gpt-evolution/)*,* [*3*](https://medium.com/@dlaytonj2/chatgpt-how-we-got-to-where-we-are-today-a-timeline-of-gpt-development-f7a35dcc660e)*,* [*4*](https://timelines.issarice.com/wiki/Timeline_of_ChatGPT)*,* [*5*](https://blog.risingstack.com/state-of-openai-gpt-models/)*,* [*6*](https://www.linkedin.com/pulse/openai-gpt-chatgpt-timeline-2022-feb-2026-sanjana-r-pmp-cspo--pxfoc)*,* [*7*](https://en.wikipedia.org/wiki/ChatGPT)*,* [*8*](https://www.ai-toolbox.co/chatgpt-models/chatgpt-models-explained-complete-comparison-2026)*,* [*9*](https://help.openai.com/en/articles/9624314-model-release-notes)*,* [*10*](https://mysummit.school/blog/en/chatgpt-review-2026/)*,* [*11*](https://en.wikipedia.org/wiki/GPT-5.4)*,* [*12*](https://www.gradually.ai/en/chatgpt-versions/)*]*

***Summary of Retired Models (February 13, 2026)***

*As of early 2026, OpenAI officially retired the older series from the main ChatGPT interface to focus on the GPT-5 family: [*[*1*](https://help.aiprm.com/hc/en-us/articles/37917157646865-Where-are-my-ChatGPT-models)*,* [*2*](https://www.designveloper.com/blog/chat-gpt-4-vs-5/)*,* [*3*](https://www.itp.net/ai-automation/openai-to-retire-gpt-4o-and-legacy-models-from-chatgpt-in-february-2026)*]*

- ***Retired:*** *GPT-4o, GPT-4.1, GPT-4.1 mini, o4-mini, and original GPT-5.*
- ***Current Setup (Spring 2026):*** *GPT-5.3 Instant (default), GPT-5.4 Thinking, GPT-5.4 Pro. [*[*1*](https://www.ai-toolbox.co/chatgpt-models/chatgpt-models-explained-complete-comparison-2026)*]*

2. Results section: Statistical Analysis Issues
• Issue A: You report p-values for pairwise comparisons (e.g., "p=0.015" for ChatGPT vs. Perplexity on GFOG) but do not state whether you applied Bonferroni correction (mentioned in Methods) to all pairwise tests. With 3 models, there are 3 comparisons per metric. At α=0.05, Bonferroni-adjusted significance would be p < 0.0167. Your reported p=0.015 would be significant, but p=0.005 would also be significant. However, some comparisons (e.g., Table 2, ChatGPT vs. Perplexity, FKGL p=0.005) are fine. Clarify which p-values are corrected.

**Dear Reviewer, thank you for this important and insightful comment.**

We confirm that Bonferroni correction was applied for all pairwise comparisons, as stated in the Methods section. Given that three pairwise comparisons were performed, the adjusted significance threshold was set at p < 0.0167.

To improve clarity and avoid any ambiguity, we have revised the manuscript as follows:

- The Statistical Analysis section has been updated to explicitly state the Bonferroni-adjusted significance threshold.
- Table footnotes have been revised to clarify that the reported p-values are unadjusted, and that statistical significance was interpreted according to the Bonferroni-adjusted threshold.

### We believe these revisions enhance the transparency and rigor of the statistical reporting. Thank you again for your valuable contribution. *Additional information for the Statistical Analysis section:*

*For all statistical analyses, a p-value of less than 0.05 was considered statistically significant. For post hoc pairwise comparisons, Bonferroni correction was applied; given that three pairwise comparisons were performed, the adjusted significance threshold was set at p < 0.0167. Reported p-values represent unadjusted values, and statistical significance was interpreted according to the Bonferroni-adjusted threshold.*

*Added below each of the first 4 tables:*

*Pairwise comparisons were performed using the Mann–Whitney U test with Bonferroni correction. Reported p-values are unadjusted; statistical significance was interpreted using a Bonferroni-adjusted threshold of p < 0.0167. Bold p-values indicate statistically significant results.*

• Issue B: You state "statistically significant difference was found between the median readability values of all answers and the sixth-grade readability" (p<0.001). What statistical test was used for this? It appears you compared a single median value against a fixed threshold (6.0). Was this a one-sample Wilcoxon signed-rank test? This must be explicitly stated.

**Dear Reviewer, thank you for this valuable methodological comment.**

We confirm that comparisons between the observed readability scores and the recommended sixth-grade readability level (grade 6.0) were performed using the one-sample Wilcoxon signed-rank test.

To improve clarity, we have explicitly stated this in the Statistical Analysis section of the revised manuscript.

### Thank you for helping us enhance the methodological transparency of our study. Additional information for the Statistical Analysis section:

*Comparisons among the three AI models were performed using the non-parametric Kruskal–Wallis test. When statistically significant differences were detected, pairwise comparisons were conducted using the Mann–Whitney U test with Bonferroni correction to control for multiple comparisons. Additionally, comparisons between the observed readability scores and the recommended sixth-grade readability level (grade 6.0) were performed using the one-sample Wilcoxon signed-rank test.*

### • Issue C: Table 5 (correlation) reports many coefficients >0.9 (e.g., JAMA vs. mDISCERN = 0.934). Such high correlations suggest potential collinearity or that scales measure nearly identical constructs. This should be discussed as a limitation. Dear Reviewer, we sincerely appreciate your valuable evaluation and attention. These scales often include criteria such as authorship, publication date, or references. This leads to similarities between scales. We have added the following to the limitations section regarding the situation you mentioned:

*Another limitation that can be highlighted is that although our study included scales used in studies with similar methodologies in the literature, it resulted in high correlation levels among scales that question similar criteria. Using scales that question different criteria in future studies will prevent this situation.*


3. Material and Methods :Missing Essential Information
• Issue A: You mention "two independent researchers (Y.E. and E.O.)" but these initials are not defined in the manuscript (no author list provided). Are these authors or external reviewers? Clarify.

Dear Reviewer, we sincerely appreciate your valuable attention. These individuals are the authors of the study. We have clarified this situation as follows:

*For quality and reliability assessments, inter-observer agreement between the two independent evaluators [Y.E. and E.O.], who were also the authors of the study, was evaluated using the Intraclass Correlation Coefficient (ICC).*


• Issue B: No information on inter-observer agreement calculation beyond ICC values. What was the confidence interval for each ICC? What constitutes "good to excellent"? Provide benchmarks (e.g., <0.5 poor, 0.5-0.75 moderate, >0.75 good).

Dear Reviewer, thank you for this valuable comment.

We have revised the manuscript to improve the reporting of inter-observer agreement. Specifically:

The Statistical Analysis section now states that ICC values were calculated with 95% confidence intervals.

We have also added standard interpretative benchmarks for ICC values (<0.50 poor, 0.50–0.75 moderate, 0.75–0.90 good, >0.90 excellent).

These additions enhance the clarity and methodological transparency of our reliability analysis.

### Thank you for your helpful suggestion. Additional information for the Statistical Analysis section:

*Correlations between quality and reliability metrics were assessed using Spearman’s rank correlation coefficient. For quality and reliability assessments, inter-observer agreement between the two independent evaluators (Y.E. and E.O.), who were also the authors of the study, was evaluated using the Intraclass Correlation Coefficient (ICC)* ***with 95% confidence intervals (CIs). ICC values were interpreted as follows: <0.50 poor, 0.50–0.75 moderate, 0.75–0.90 good, and >0.90 excellent reliability.***

Correction in the Result section:

*Inter-rater agreement was high in quality and reliability assessments. Intraclass Correlation Coefficient values were as follows: mDISCERN 0.761, JAMA 0.876, GQS 0.795, and EQIP 0.999, indicating good to excellent reliability according to predefined ICC interpretation thresholds.*

• Issue C: You state "browser history and cookies were completely cleared" and used "Incognito" mode. However, you did not mention using a VPN to control for geographic variation in search results. Since Google Trends data showed varying interest by country (Philippines, Thailand, USA), server location could bias results. This is acknowledged in Limitations, but it should also be stated in Methods that no VPN was used.

Dear Reviewer, we sincerely appreciate your valuable evaluation. We added this to the methods section as follows:

*The research was conducted without the use of a VPN, via Google's "Incognito Mode" tab, to minimize the impact of historical data.*


4.Abstract and Discussion: Writing and Clarity Problems
• Issue A: Abstract is overly dense. The sentence beginning "While AI platforms exhibit high potential..." is 58 words long. Break it into shorter sentences.

Dear Reviewer, we sincerely appreciate your valuable attention.
Actually, that section contains two sentences. The first sentence consists of 29 words and the second of 30 words. Following your suggestion, these two sentences have been simplified and presented as four sentences:

*Artificial intelligence platforms have been observed to exhibit high potential in the production of medical information. However, linguistic barriers exceeding sixth-grade reading comprehension, along with reliability limitations of current models, prevent them from replacing professional medical consultation. Perplexity has been found superior in terms of academic quality, while ChatGPT has been found superior in terms of readability. Nevertheless, positioning these systems as complementary "secondary consultation mechanisms" supporting physician oversight in clinical decision-making processes is critically important for patient safety.*


• Issue B: You introduce an undefined acronym "MPSs Learning Achievement" in the Discussion (first paragraph). This appears to be an error. Delete.
Dear Reviewer, we sincerely appreciate your valuable attention.
This section has been deleted based on your suggestions.

• Issue C: The phrase "MPS [MPSsive Activity Syndrome]" appears in the Conclusion. This is incorrect. MPS stands for Myofascial Pain Syndrome throughout the manuscript. Remove the erroneous expansion.
Dear Reviewer, we sincerely appreciate your valuable attention.
This section has been deleted based on your suggestions.

• Issue D: In the Introduction, page 2: "limited range of motionan decreased functional capacity" – missing space and likely typo ("and").

Dear Reviewer, we sincerely appreciate your valuable attention. We fixed this situation as follows:

Clinically, regional pain is often accompanied by muscle spasm, limited range of motion, and decreased functional capacity; this can significantly affect patients' daily activities and quality of life [2].


• Issue E: Results section: "The research focused on eighteen key keywords" – redundant. "Eighteen keywords" suffices.
Dear Reviewer, we sincerely appreciate your valuable attention. We fixed this situation as follows:

*Eighteen keywords were evaluated in the study.*

5. Discussion section: Interpretation and Discussion Weaknesses
• Issue A: You state "ChatGPT exhibits the most optimized performance in terms of readability" but readability is not necessarily a virtue if it comes at the cost of accuracy. ChatGPT had lower quality/reliability scores. This trade-off is mentioned but not adequately explored. Add a paragraph discussing whether easier readability might actually increase risk if the content is less reliable.
Dear Reviewer, we sincerely appreciate your valuable attention. We added this situation as follows:

*While our study showed ChatGPT exhibited higher readability, it doesn't guarantee high accuracy. In fact, when AI writes very fluently and clearly, it can create a 'false sense of authority' by making misinformation appear professional and trustworthy. This is a significant risk because people tend to believe and follow advice that is easy to read, even if it is wrong. If a text is easy to understand but has low reliability, high readability actually helps spread misinformation more effectively, making it more dangerous.*

• Issue B: You cite a study on fibromyalgia (reference 35) but do not mention whether those findings align with or contradict your own. Specifically, that study found Gemini offered higher quality for fibromyalgia, whereas you found Perplexity superior for MPS. Discuss possible reasons (different condition, different version of models).


Dear Reviewer, we sincerely appreciate your valuable attention. While our study found Perplexity to be superior for Myofascial Pain Syndrome (MPS), these results contrast with the study by reference 35 (**Zure and Menekşeoğlu (2025))**, which found Gemini to be of higher quality for fibromyalgia. This difference is likely due to the nature of the three conditions; fibromyalgia is a widespread, systemic condition, whereas MPS is a more localized musculoskeletal issue, and AI models may process these distinct clinical profiles with varying degrees of accuracy. Furthermore, the versions of the AI models used likely differ between the two studies. Because AI technology evolves rapidly, model updates can significantly change performance over time, meaning the version of Gemini or Perplexity tested in the fibromyalgia study may have performed differently than the versions evaluated in our MPS research. Finally, while the cited study on fibromyalgia utilized only 10 questions for its assessment, our study utilized 18 questions. This larger set of questions provides a more comprehensive sample, allowing for a more detailed and robust analysis of the information generated by the AI models.
We added this situation as follows:


*In the same study, the authors found that Gemini, rather than Perplexity, produced the highest quality data, unlike in our study. This can be explained by the fact that FMS, unlike MPS, is a widespread, systemic condition, the artificial intelligence models used in their studies are different, and the results were expressed with a smaller sample size compared to our study.*

### • Issue C: The conclusion that AI should be a "secondary advisory mechanism" is reasonable but vague. What specific safeguards do you propose? (e.g., mandatory disclaimer labels, AI-generated text being reviewed by a human clinician before patient access, integration with EHRs?) We thank the reviewer for the specific suggestions regarding safeguards. While we considered integration with EHRs, we decided not to emphasize it due to significant concerns regarding patient data privacy and information security. Instead, we have updated the manuscript to focus on more immediate and secure safeguards: (1) Mandatory disclaimer labels, (2) a 'clinician-in-the-loop' review to verify AI content before patient access, and (3) source transparency requiring links to evidence-based literature. We believe these measures provide robust protection without compromising sensitive patient data. We added this situation as follows: *To ensure AI acts as a reliable secondary advisory mechanism, specific safeguards are necessary. We propose mandatory disclaimer labels to distinguish AI from professional advice, a 'clinician-in-the-loop' validation process to verify content accuracy before it reaches patients, and transparent citations to evidence-based literature. These measures are essential to mitigate 'fluency bias,' ensuring that high readability does not lead patients to trust low-quality or unverified medical information.* 6. Minor Concerns Suggested Title Revision Current title is very long (20+ words). Consider shortening while preserving key elements: Option A: "Readability, Quality, and Reliability of AI-Generated Information on Myofascial Pain Syndrome: A Comparative Analysis of ChatGPT, Gemini, and Perplexity" Option B: "Are AI Chatbots Readable and Reliable for Myofascial Pain Syndrome Information? A Comparison of Three Large Language Models" We appreciate the reviewer’s suggestion regarding the title length. We agree that a more concise title improves clarity. Among the proposed options, we have selected Option A: 'Readability, Quality, and Reliability of AI-Generated Information on Myofascial Pain Syndrome: A Comparative Analysis of ChatGPT, Gemini, and Perplexity'. This version effectively preserves all key elements of our research—including the specific metrics and models used—while significantly improving brevity and professional tone.

*We would like to thank all our reviewers for their valuable opinions and suggestions. We learned a lot from you..*
